# Fragile X astrocytes induce developmental delays in dendrite maturation and synaptic protein expression

**DOI:** 10.1186/1471-2202-11-132

**Published:** 2010-10-18

**Authors:** Shelley Jacobs, Meera Nathwani, Laurie C Doering

**Affiliations:** 1 Department of Pathology & Molecular Medicine, McMaster University, 1200 Main Street West, Hamilton, Ontario, L8N 3Z5, Canada

## Abstract

**Background:**

Fragile X syndrome is the most common inherited form of mental impairment characterized by cognitive impairment, attention deficit and autistic behaviours. The mouse model of Fragile X is used to study the underlying neurobiology associated with behavioral deficiencies. The effect of Fragile X glial cells on the development of neurons has not been studied. We used a co-culture technique in combination with morphometrics on immunostained neurons to investigate the role of astrocytes in the development delays associated with hippocampal neuron development.

**Results:**

We found that hippocampal neurons grown on Fragile X astrocytes exhibited a significant difference from the neurons grown with normal astrocytes after 7 days in vitro for many parameters including increases in dendritic branching and in area of the cell body. However, after 21 days in culture, the neurons grown on Fragile X astrocytes exhibited morphological characteristics that did not differ significantly from the neurons grown on normal astrocytes. With antibodies to the pre-synaptic protein, synapsin, and to the excitatory post-synaptic protein, PSD-95, we quantified the number of developing excitatory synapses on the dendrites. In addition to the delays in dendritic patterning, the development of excitatory synapses was also delayed in the hippocampal neurons.

**Conclusions:**

These experiments are the first to establish a role for astrocytes in the delayed growth characteristics and abnormal morphological features in dendrites and synapses that characterize the Fragile X syndrome.

## Background

As the central nervous system (CNS) develops numerous events must occur in a highly regulated manner to create the intricate organization of neural networks that control the mature brain. One of the key players recognized in the guidance of neuron development is the astrocyte [1-5]. As such, abnormal or 'diseased' astrocytes are now known to be prominent factors in the neurobiology of a number of developmental diseases of the CNS including Fragile X Syndrome (FXS) [3,6-8].

FXS is the most common inherited form of mental retardation, affecting approximately 1/2500 children [9]. Children with FXS suffer from a number of behavioural deficiencies including: mild to severe cognitive impairment, hyperactivity, attention deficit, susceptibility to seizures, motor disorders and autistic behaviours [10]. Underlying neurobiological abnormalities in FXS are recognized in the form of altered dendritic growth, abundant immature dendritic spines and inappropriate synaptic development [11-13]. The defects in individuals with FXS can be attributed to a mutation in the Fragile X Mental Retardation 1 (*FMR1*) gene causing gene silencing and a lack of the protein product, Fragile X Mental Retardation Protein (FMRP) [14].

The *Fmr1 *knockout (-/-) mouse model of FXS is well accepted as an appropriate model in which to study the neurobiology of FXS [15]. The *Fmr1*-/- mouse has been shown to demonstrate behavioural qualities similar to those seen in individuals with FXS, including: susceptibility to seizures, hyperactivity and learning impairments [15-17]. Additionally, the neurons of *Fmr1*-/- show similarly abnormal dendritic spine morphology and altered synaptic function [18,19], as documented in humans with FXS [11].

Contrary to the current belief that FMRP is only expressed in neurons, recent work in our lab revealed that FMRP is also expressed in cells of the glial lineage in the *Fmr1*-/- mouse [20]. Based on these findings we began our investigation into the role of astrocytes in the development of the abnormal neurobiology of FXS. Previously, we used the *Fmr1*-/- mouse model to investigate the role of astrocytes in the development of the abnormal neuronal characteristics seen in FXS [21]. In short, we found that Fragile X astrocytes were not able to support normal neuron growth and contributed to the abnormal neuronal phenotype seen in the Fragile X mouse. However, in that series of experiments, we focused on the early development of neurons, solely evaluating the effect of null astrocytes on the neurons after seven days in culture. Given that FXS is a developmental disorder, we wondered if neurons were prevented from normal maturation when exposed to signaling from the Fragile X astrocytes. If so, is it possible that neurons exposed to Fragile X astrocytes could recover from developmental delays? If true, this information would foster insight into the potential for astrocyte-directed therapeutic interventions as a preventative measure against the neuronal abnormalities underlying FXS. Such is the basis for the following experiments in which we investigated the possibility of an astrocyte-mediated a delay in neuron development using detailed morphometric and synaptic protein analyses combined with immunocytochemistry.

## Methods

### Animals

The FMRP knock-out FVB.129P2(B6)-*Fmr1^tm1Cgr ^*[15] and wild-type (WT) mice used for these experiments were housed and bred at the McMaster University Central Animal Facility. All experiments were completed in accordance with the guidelines set out by the Canadian Council on Animal Care and were approved by the McMaster Animal Research Ethics Board.

### Cell Culture

Primary hippocampal neurons with astrocytes were grown in co-culture conditions as detailed previously by our lab [22]. Briefly, cortical astrocytes were isolated from post-natal day (PND) 0-1 pups and grown on Poly-L-Lysine (1 mg/ml, Cat. No. P1399, SIGMA-ALDRICH, Oakville, Canada) and Laminin (0.1 mg/ml, Cat. No. 23017-015, Invitrogen, Burlington, Canada) coated coverslips in MEM (Cat No. 11095-080, Invitrogen, Burlington, Canada) supplemented with 6% glucose and 10% horse serum (Cat No. 16050-122, Invitrogen, Burlington, Canada), for one week. Primary hippocampal neurons were then isolated from embryonic day 17 (day of sperm plug counted as PND1) animals and seeded above the astrocytes and maintained in MEM supplemented with N2 (Cat No. 17502-048, Invitrogen, Burlington, Canada), sodium pyruvate (Cat No. 11360-070, Invitrogen, Burlington, Canada) and 6% glucose, for the duration of the experiments. Hippocampal neurons isolated from WT animals were grown with astrocytes, from either WT or *Fmr1*-/- mice, in culture for 7, 14 or 21 days. These cultures were grown in culture for 7, 14 or 21 days. A total of three independent cultures per condition (genotype+days *in vitro *(DIV)) were completed.

### Immunocytochemistry

Following removal of the media, the cells were fixed with 4% PFA in PBS or ice cold (-20°C) acetone for 15 or 20 minutes, respectively. After three washes with PBS, and the application of 0.1% Triton X-100 where necessary, non-specific binding was blocked with 1% bovine serum albumin (BSA) for 30 minutes. Primary antibodies were applied to the coverslips and incubated overnight at 4°C. The second day, following washes with PBS, secondary antibodies were incubated with the cells for 3 hours at room temperature. Following a final set of washes with PBS and distilled water, the coverslips were mounted onto slides with Vectashield fluorescent mounting medium (Vector Labs, Burlington, Canada) containing DAPI to stain the nuclei.

The following antibodies, diluted in 1% BSA, were used: chicken anti-Map2 (Microtubule Associated Protein -2, 1:20,000, Neuromics, Edina, MN), guinea pig anti-synapsin (1:1000, Synaptic Systems, Goettingen, Germany), mouse monoclonal anti-PSD-95 (Post-Synaptic Density protein-95, Clone 6G6-1C9, 1:200, Millipore, Temecula, CA), anti-mouse AlexaFluor 594 (1:1500, Invitrogen, Burlington, Canada), anti-guinea pig FITC (1:200, Jackson ImmunoResearch, West Grove, PA) and anti-chicken FITC (1:100, Jackson ImmunoResearch, West Grove, PA).

### Image acquisition

Images were acquired using a Zeiss Axioskop 2 epi-fluorescence microscope and Axiovision (v4.6) image acquisition software.

### Dendritic arbor morphology

**Figure 1:**
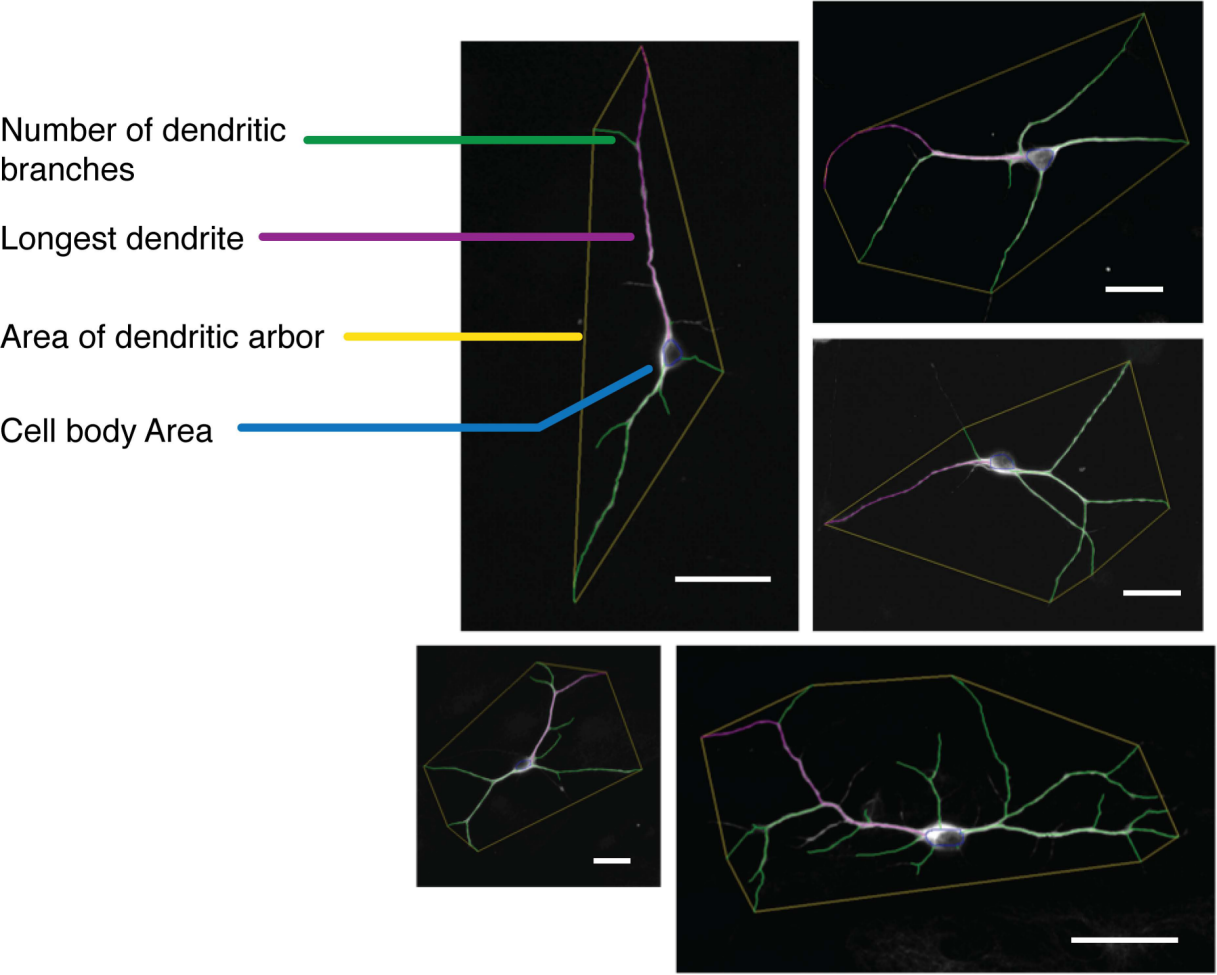
**A selection of representative images of neurons overlaid with skeletons as generated by Neuronmetrics™**. The parameters measured in these experiments are indicated on the left of the images: number of dendritic branches (green), longest dendrite (purple), area of dendritic arbor (bounded by yellow), and cell body area (blue). Scale bars represent 50 μm.

**Figure 2:**
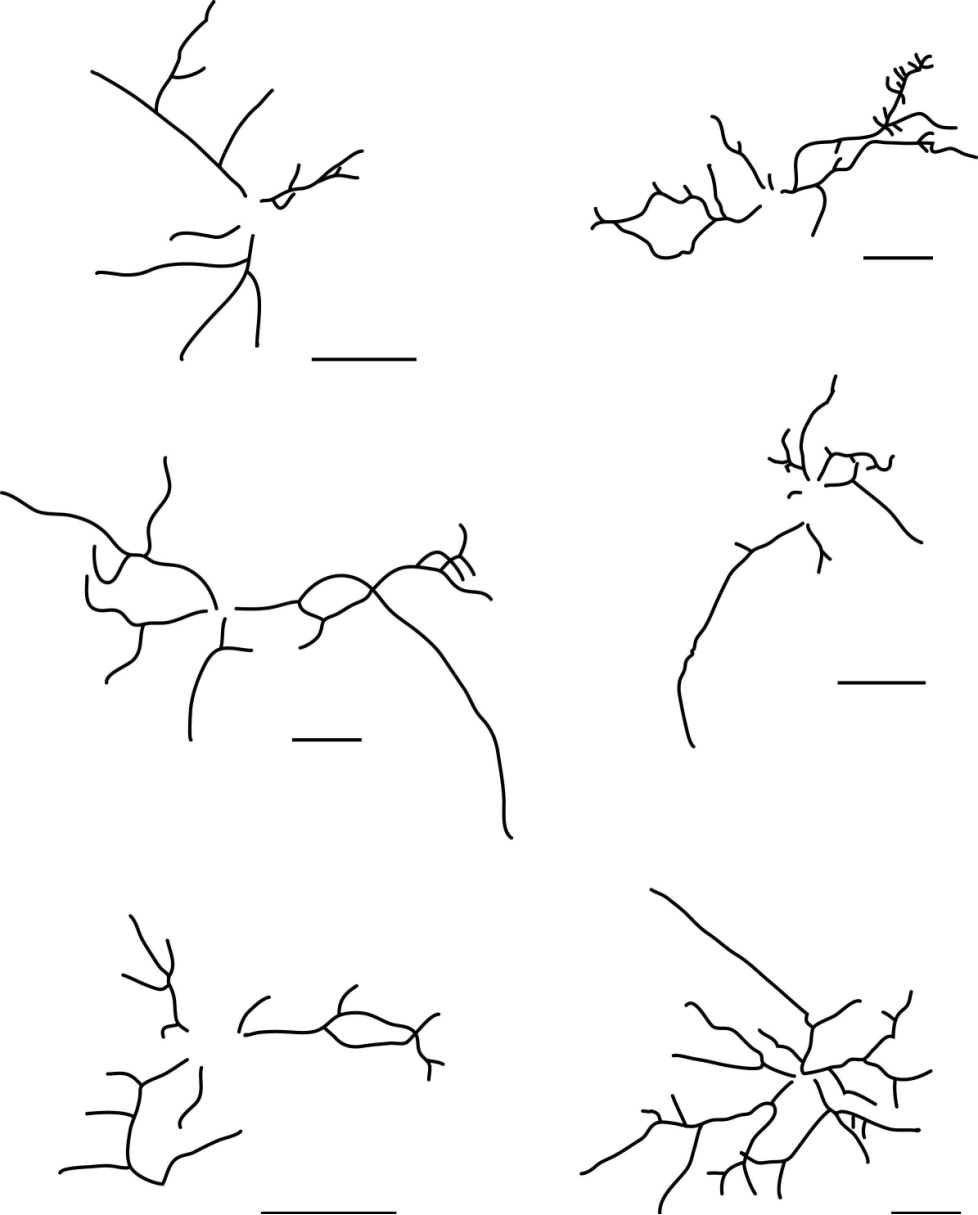
**A selection of digitized skeletons, generated by Neuronmetrics™, used to generate the measurements of the dendritic arbors**. Scale bars represent 50 μm.

Morphological measurements were conducted using Neuronmetrics™ http://www.ibridgenetwork.org/arizona/UA07-56-Neuronmetrics[23], a plug-in for Image J http://rsbweb.nih.gov/ij/. The following parameters were evaluated: number of dendritic branches, length of the longest primary dendrite, and the area covered by the dendritic arbor. For each independent culture, 50 isolated MAP2(+) neurons were selected and analyzed for morphology. Once each neuron image was converted to a black and white TIFF file, the images were processed through Neuronmetrics™ to generate pixilated skeletons and to obtain the morphological measurements listed above. Examples of representative neurons overlaid with digitized skeletons and area demarcations generated by Neuronmetrics™ are shown in Figure 1. Representative skeletons used by Neuronmetrics™ for morphological measurements are shown in Figure 2.

### Cell body measurements

For each of the above neurons the area of the cell body was also measured, by tracing around the cell body using Image J.

### Synaptic puncta analysis

Synapses were identified by the co-localization of the pre- (synapsin) and post-synaptic (PSD-95) puncta, using a custom written plug-in for Image [24]. After selecting a 50 μm segment along the dendrite, the image was processed using the above plug-in. Briefly, low frequency background from each channel (red and green) of the image was removed with the rolling ball background subtraction algorithm. Then, the puncta in each single-channel was 'masked' by thresholding the image so that only puncta remained above threshold. Puncta were then identified in each channel by the "Particle Analyzer" plug-in for Image J. Co-localization of the puncta in each channel was indentified when the distance between the centers of the two puncta was less than the radius of the larger puncta. The number of co-localized puncta was then recorded as synapses. For each independent culture one hundred 50 μm-long sections along the dendrites were evaluated for synaptic protein expression.

### Statistical Analyses

Statistics were performed using SPSS version 17, with an alpha level set to 0.05. All data are presented as mean ± SEM. Where the data had significantly different variances (as determined by Levene's test for equal variances), and deviated significantly from a normal distribution (as determined by the Shapiro-Wilk test for normality, Mann-Whitney U (two-tailed) tests were used.

## Results

### Morphological Analysis

**Figure 3:**
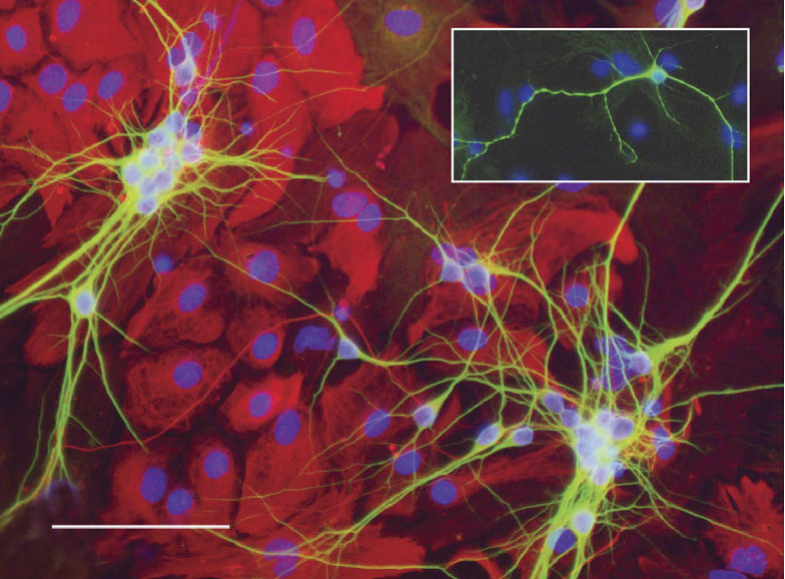
**Hippocampal neurons in co-culture at 7*DIV***. WT primary embryonic hippocampal neurons (MAP2, green) on top of a monolayer of WT primary cortical astrocytes (GFAP, red). Nuclei are stained with DAPI (blue). Scale bar represents 100 μm. Inset is an example of an isolated neuron that would be selected for morphometric analysis.

In order to visualize the dendritic arbors of the neurons in co-culture, immunofluorescence using an antibody directed against the dendrite marker MAP2 was used [25]. Figure 3 provides an example of the hippocampal neuron- astrocyte co-culture at 7 *DIV*, stained with MAP2 (green) to enable the visualization of the dendritic arbors. In the same image the astrocytes are stained with an antibody directed against Glial Fibrillary Acidic Protein (GFAP, red), and the nuclei are stained with DAPI (blue). Upon examination of the cultures, no obvious differences could be seen between clusters of neurons grown on *Fmr1*-/- compared to those grown on WT astrocytes. To perform the morphometric analysis, isolated neurons were selected at random from the coverslip (Figure 3, inset).

**Figure 4:**
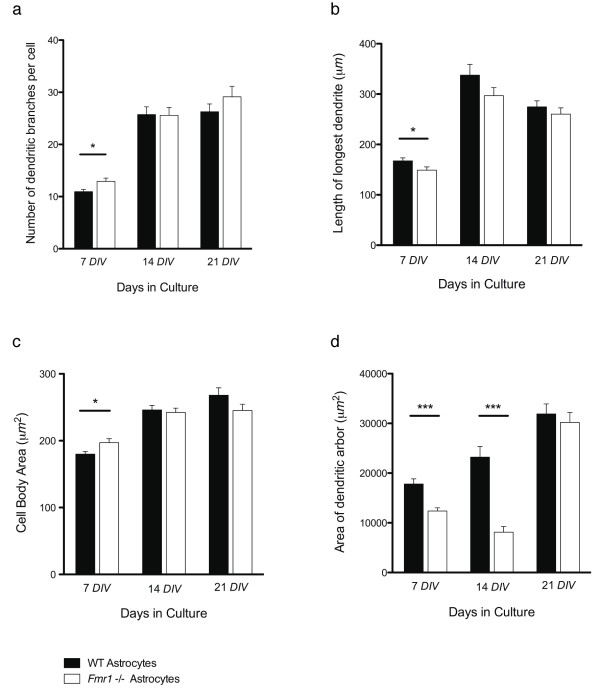
**Analysis of the morphological measurements generated by Neuronmetrics™: a, number of dendritic branches per cell; b, length of the longest dendrite; c, cell body area; d, area of dendritic arbor**. In each graph the number of days *in vitro *(*DIV*) is represented along the x-axis. Data from neurons grown on WT astrocytes are represented by the solid bars, and from neurons grown on *Fmr1*-/- astrocytes are represented by the white bars. Data are presented as mean ± s.e.m. of 150 neurons from three independent experiments. *, significantly different at P < 0.05; ***, significantly different at P < 0.001; Mann Whitney U Tests (two-tailed).

At 7 *DIV*, the number of dendritic branches per cell was significantly (p = 0.045; Mann Whitney U = 9745.50; F = -2.007) increased in the neurons grown on *Fmr1*-/- astrocytes (12.90 ± 0.62) compared to those grown on WT astrocytes (10.93 ± 0.44)(Figure 4a). Branching increased over time in neurons grown on both *Fmr1*-/- and WT astrocytes. However, by 14 *DIV *there was no significant difference in the number of dendritic branches between neurons grown on *Fmr1*-/- astrocytes versus those grown on WT astrocytes (*Fmr1*-/-, 25.62 ± 1.49; WT, 25.69 ± 1.49; p = 0.794; Mann Whitney U = 11054.00; F = -0.261). This was also true for the cells measured at 21 *DIV *(*Fmr1*-/-, 29.13 ± 2.02; WT, 26.27 ± 1.48; p = 0.818; Mann Whitney U = 11077.00; F = -0.230).

The length of the longest dendrite was also significantly different at 7 *DIV*. At 7 *DIV*, neurons grown on *Fmr1*-/- astrocytes exhibited significantly (p = 0.019; Mann Whitney U = 9491.00; F = -2.342) lower values (148.93 ± 6.47) of their longest dendrites compared to neurons grown on WT astrocytes (167.40 ± 6.47)(Figure 4b). The length of the longest dendrite increased at 14 *DIV*, and decreased slightly at 21 *DIV*, for neurons grown on both *Fmr1*-/- and WT astrocytes. No significant difference in length of the longest dendrite was observed at 14 *DIV *(*Fmr1*-/-, 295.94 ± 16.00; WT, 338.86 ± 21.14; p = 0.261; Mann Whitney U = 10335.00; F = -1.124) or 21 *DIV *(*Fmr1*-/-, 263.78 ± 12.29; WT, 274.78 ± 11.97; p = 0.229, Mann Whitney U = 10205.00; F = -1.203).

At 7 *DIV*, but not 14 or 21 *DIV*, the cell body area was significantly (p = 0.047; Mann Whitney U = 9754.50; F = -1.991) increased in neurons grown on *Fmr1*-/- astrocytes (197.10 ± 5.87) compared to those grown on WT astrocytes (179.85 ± 3.95)(Figure 4c). The cell body area was increased in neurons grown on both *Fmr1*-/- and WT astrocytes 14 *DIV*, and only slightly so at 21 *DIV*. Once again, there was no significant difference in this morphological parameter between neurons grown on *Fmr1*-/- or WT astrocytes at 14 *DIV *(*Fmr1*-/-, 242.27 ± 6.45; WT, 246.02 ± 6.66; p = 0.840; Mann Whitney U = 11098.00; F = -0.202) or 21 *DIV *(*Fmr1*-/-, 245.13 ± 9.44; WT, 268.05 ± 11.08; p = 0.228; Mann Whitney U = 10344.50; F = -1.205).

The area of the dendritic arbor was significantly decreased in neurons grown on *Fmr1*-/- astrocytes compared to those grown on WT astrocytes at both 7 *DIV *(*Fmr1*-/-, 12373.57 ± 654.79; WT, 17800.08 ± 1038.79; p = 0.000; Mann Whitney U = 8278.00; F = -3.956) and 14 *DIV *(*Fmr1*-/-, 8116.14 ± 1126.15; WT, 23209.23 ± 2153.68; p = 0.000; Mann Whitney U = 6001.00; F = -6.987) (Figure 4d). The area of the dendritc arbor increased with time in culture for neurons grown on WT astrocytes. At 21 *DIV *the area of the dendritic arbor of neurons grown on *Fmr1*-/- astrocytes also increased, and was comparable to that of the neurons grown on WT astrocytes. The difference was not significant at this point (21 *DIV*) in culture (*Fmr1*-/-, 30192.00 ± 1995.85; WT, 31900.05 ± 2020.96; p = 0.539; Mann Whitney U = 10789.00; F = -0.614).

Therefore, by the morphological parameters evaluated in these experiments, it appears that the growth of the neurons early in development (i.e., 7 *DIV*) is altered when grown on *Fmr1*-/- astrocytes, but these abnormal characteristics disappear with time. By 14 *DIV*, three out of four of the morphological characteristics we studied were no longer significantly altered in neurons grown on *Fmr1*-/- astrocytes. The last parameter, the area of the dendritic arbor, had also reached levels comparable to values seen in neurons grown with WT astrocytes by 21 *DIV*.

**Figure 5:**
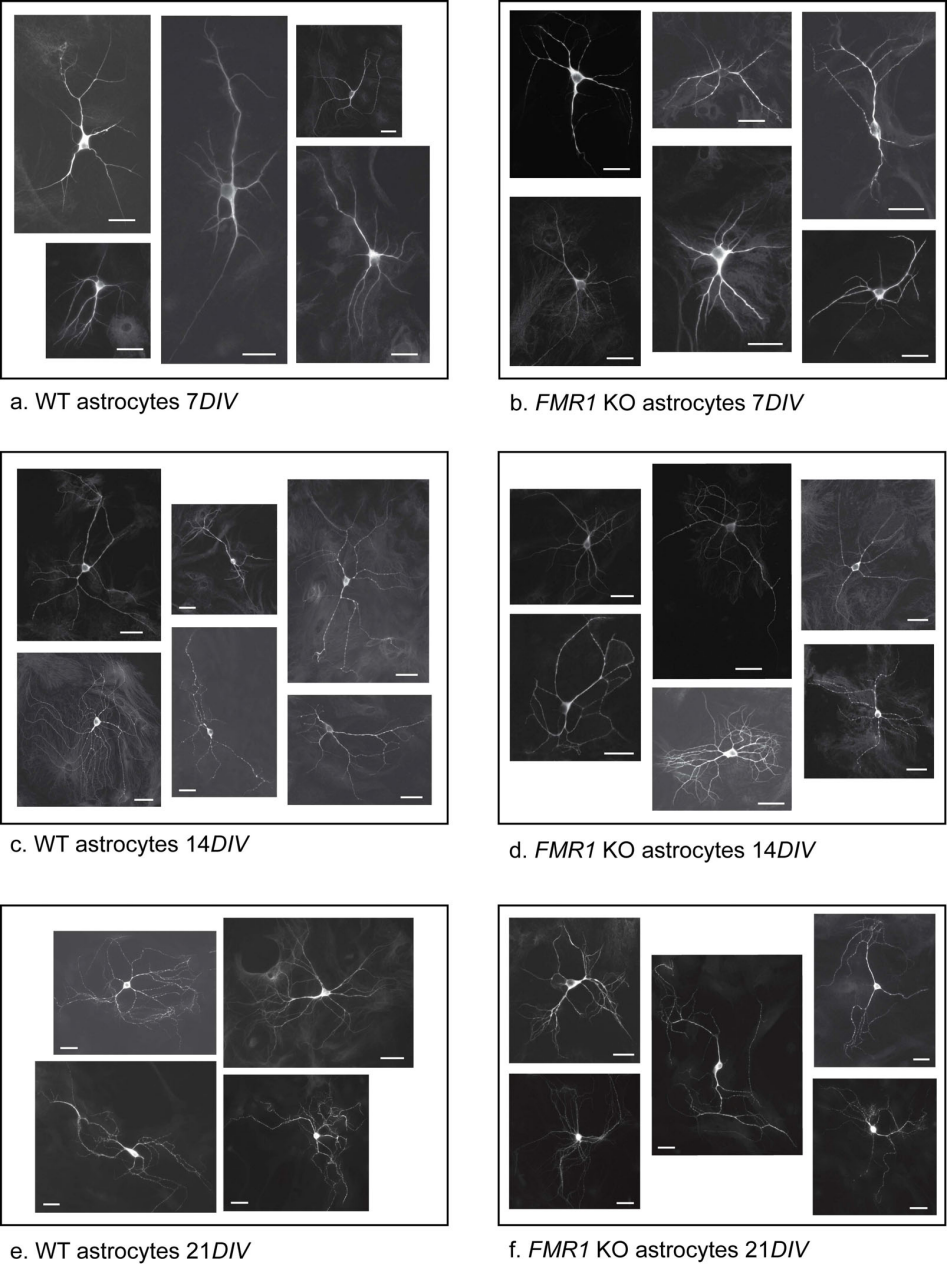
**Examples of MAP2 immunofluorescent in neurons used for morphometrics**. MAP2 staining identifies the cell bodies and dendrites. Scale bars equal 50 μm.

Representative images of the parameters measured in Figure 4 are shown in the panel composite of Figure 5.

### Excitatory Synapse Analysis

**Figure 6:**
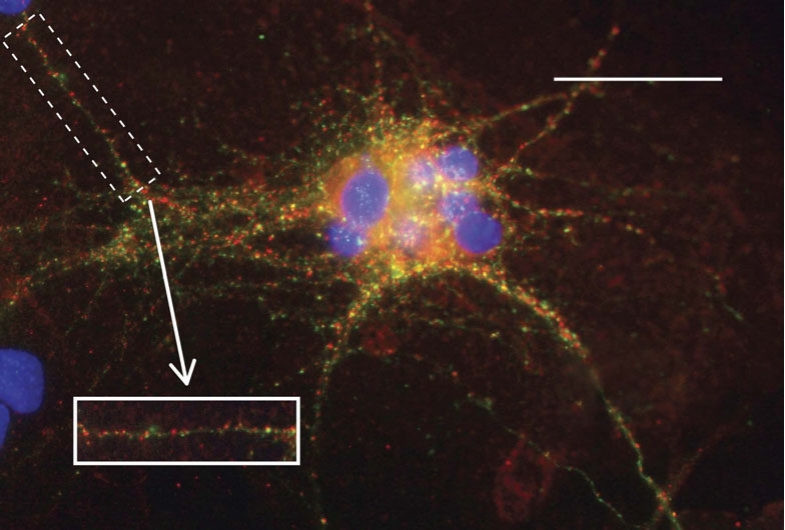
**Excitatory synaptic protein expression in hippocampal neurons in co-culture**. Hippocampal neurons are stained with antibodies to the pre-synaptic protein, synapsin (green) and the excitatory post-synaptic protein, PSD-95 (red). Nuclei are stained with DAPI (blue). Scale bar represents 100 μm. Inset is an example of a 50 μm segment that would be used for excitatory synapse quantification.

Excitatory synapses were visualized using immunofluorescence. An antibody directed at synapsin, a synaptic vesicle protein known to be involved in neurotransmitter release [26], was used to identify pre-synaptic components. Excitatory synapse post-synaptic regions were detected using an antibody directed at the post-synaptic protein, PSD-95, that is known to be part of the post-synaptic density complex found at excitatory synapses [27]. Double-labeling of these pre- and post-synaptic proteins was followed by the quantification of co-localized pre- and post-synaptic puncta using a plug-in for Image J. Figure 6 provides an example of a network of dendrites in the hippocampal neuron culture double-stained for synapsin (green) and PSD-95 (red). To perform the quantification, segments of dendrites that were 50 μm long were isolated and processed for co-localization analysis (Figure 6, rectangular region and inset).

**Figure 7:**
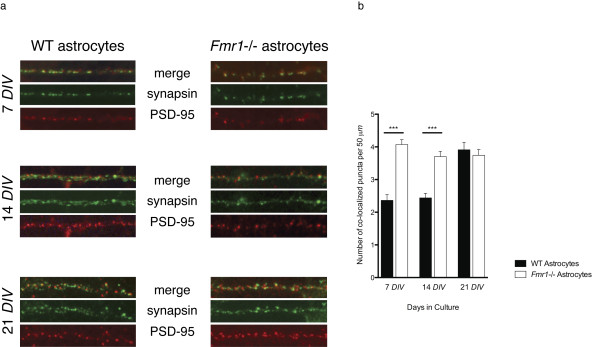
**Quantification of synaptic protein co-localization a. Excitatory synaptic protein expression in segments of dendrites from neurons grown for 7, 14 or 21 *DIV *on either WT astrocytes (left panel) and on *Fmr1*-/- astrocytes (right panel)**. Each set of three images represents the same 50 μm segment visualized with immunofluorescence directed at pre- and post-synaptic proteins (top, merge), pre-synaptic synapsin (green, middle), and post-synaptic PSD-95 (red, bottom). **b**. From similar 50 μm segments, the number of synapses (i.e., instances of co-localized puncta) per 50 μm were quantified. The number of days in culture (*DIV*) are indicated along the x-axis. The solid bars are data from neurons grown on WT astrocytes. The white bars are data from neurons grown on *Fmr1*-/- astrocytes. Data are presented as mean ± s.e.m., from 300 segments from three independent experiments. ***, significant at P < 0.001, Mann Whitney U tests (two-tailed).

At 7 *DIV *neurons grown on *Fmr1*-/- astrocytes had more than double the number of excitatory synapses per 50 μm compared to neurons grown on WT astrocytes (*Fmr1*-/-, 4.15 ± 0.14; WT, 1.95 ± 0.10; p = 0.000; Mann Whitney U = 20684.50; F = -11.129)(Figure 7a, b). This significant difference was maintained at 14 *DIV*; neurons grown on *Fmr1*-/- astrocytes had 53.8% more synapses per 50 μm compared to neurons grown on WT astrocytes (*Fmr1*-/-, 3.70 ± 0.17; WT, 2.40 ± 0.14; p = 0.000; Mann Whitney U = 31908.50; F = -6.229, Figure 7a, b). By 21 *DIV*, however, there was no significant difference between the number of synapses per 50 μm of dendrite for neurons grown on *Fmr1*-/- or WT astrocytes (*Fmr1*-/-, 3.68 ± 0.18; WT, 4.00 ± 0.21; p = 0.577; Mann Whitney U = 43822.50; F = -0.558). The number of synapses per 50 μm increased over time in neurons grown on WT astrocytes; whereas in neurons grown on *Fmr1*-/- astrocytes, there was a slight decrease in the number of synapses over time (Figure 7b).

Therefore, similar to the morphological characteristics we evaluated, it appears that the abnormal number of synapses found at 7 *DIV *and 14 *DIV *on neurons grown with *Fmr1*-/- astrocytes is brought to levels comparable to normal by 21 *DIV*.

## Discussion

In this study we used a co-culture procedure in which normal primary hippocampal neurons were co-cultured with normal or *Fmr1*-/- astrocytes for up to 21 days *in vitro *in order to investigate the possibility of an astrocyte-mediated delay in hippocampal neuron development. We evaluated the changes in neuron dendritic arbor morphology and excitatory synapse expression at 7, 14 and 21 *DIV*. We found that both the morphological parameters and the synaptic protein expression approached normal levels by 21 *DIV*, with three of four morphological parameters approaching normal at 14 *DIV*. Our results suggest that *Fmr1*-/- astrocytes do not completely prevent normal hippocampal neuron growth, but delay it.

The morphological results presented herein are consistent with our previously reported results for the effects of *Fmr1 *-/- astrocytes on normal primary hippocampal neurons at 7 *DIV *[21]. This finding is further supported by the earlier studies done by Lee et al. (2003). Consistent with our results, they described an increase in dendritic branching in neurons from mice lacking the *Drosophila dfmr1 *gene [28]. However, the results of the present synaptic protein expression analysis at 7*DIV*, is different from the synaptic protein analysis at 7 *DIV *of that same series of experiments. This difference could be due to the modifications of the experimental design in the present studies. First, in this study we used synapsin as our pre-synaptic protein antigen. While we would not expect that there would be a difference in the number of pre-synaptic puncta identified by the synapsin antibody versus the previously used synaptophysin antibody, this difference should be noted. Second, in the previous report we evaluated the pre- and post synaptic proteins independently whereas in the present experiments we report the co-localization of the pre-and post-synaptic proteins. It is in fact possible that the number of pre- or post- synaptic protein puncta does not correlate in a positive direction with the number of synapses. If that were the case there could be a decrease in the numbers of pre- and post-synaptic puncta independently, but there could be an increase in the number of co-localized puncta. Third, and most importantly, here we report the synaptic protein expression in segments of dendrites taken from neuron networks, whereas in the previous paper we focused on the absolute numbers of pre- and post-synaptic puncta per isolated neuron. It could be expected that synapse number will vary when comparing neural networks with isolated neurons.

### Dendritic arborization is delayed in neurons grown with *Fmr1*-/- astrocytes

The results presented here support the idea that hippocampal neurons grown with *Fmr1*-/- astrocytes reach normal maturity, but are delayed in the process. At 7 *DIV *the neurons grown on *Fmr1*-/- astrocytes had significantly different cell measurements from the neurons grown on WT astrocytes for all of the parameters we measured. However, by 21 *DIV *those neurons had morphological measurements that closely resembled those of neurons grown on normal astrocytes.

During development the growth of dendritic arbors is a highly choreographed dynamic balance between phases of extension and retraction. Cues that regulate the initiation and promote dendritic arbor development are equally as important as those that stop its elaboration [29]. There is compelling evidence suggesting that dendritic arborization is regulated by both intrinsic and extrinsic signals. Astrocytes are known to provide a number of molecules that have been shown to be involved in the growth and maturation of dendritic arbors [1-3].

In the early stages of development the neurons grown on *Fmr1*-/- astrocytes appear to be in a branching mode of growth, rather than a mode dominated by arbor extension. This suggests that the dendritic arborization is altered in a manner that would interfere with the normal appropriate wiring of the hippocampal circuits. In 2001, Cline suggested that mechanisms that enhance dendritic arbor complexity have the potential to affect the integrative properties and firing characteristics of a neuron [29]. Neurons with more densely branched arbors may receive greater numbers of synaptic input that may affect the manner in which convergent inputs influence the neuron [29]. Astrocytes are known to provide cues that can alter the development of dendritic arbors [2,3,30]. Here we show that astrocytes induce increased branching in the early stages of development of hippocampal neuron dendritic arbors *in vitro*. Thus, there is the possibility that astrocytes induce an increase in branching in the early development of hippocampal neurons in FXS, resulting in the alteration of the signaling properties of the neurons. In turn, this may affect key developmental stages when initial neural networks are being established. These alterations could contribute to the underlying abnormal neurobiology, and therefore the clinical deficiencies observed in FXS.

In addition, it is possible that astrocytes from an *Fmr1*-/- animal may be unable to appropriately regulate the establishment of the receptive field margins. Under normal conditions there are "stop" signals that inhibit the expansion of dendrites beyond the normal receptive field of the arbor [31]. Although poorly understood, some of these cues are known to originate in a contact dependent manner from the dendrites of adjacent neurons, and from contact independent means via secreted molecules (reviewed in [31]). Astrocytes present a number of signals that mediate other aspects of dendrite growth and elaboration and therefore it is possible that astrocytes may also play a role in dendritic tiling. In FXS, the 'diseased' astrocytes may inhibit the expansion of the arbor to an abnormal extent, reducing the size of the area of coverage. At later stages in development, other cues could help mediate these adverse effects, and enable the extension of the arbor. However, during that early period of inhibited expansion, irreversible damage may have occurred, thereby preventing the establishment of local circuits in a timely manner necessary for normal neural network maturation. These alterations that develop early in the dendritic arbor could contribute to the underlying abnormal neurobiology, and result in cognitive impairments.

### Excitatory synapse formation is delayed in neurons grown with *Fmr1*-/- astrocytes

The findings of this study suggest that the growth of hippocampal neurons with *Fmr1*-/- astrocytes impedes the appropriate timing of excitatory synaptic development. At 7 *DIV*, neurons grown on *Fmr1*-/- astrocytes had significantly more excitatory synapses than the neurons grown on WT astrocytes. However, by 21 *DIV *the numbers of excitatory synapses in neurons grown on *Fmr1*-/- astrocytes were equal to the quantity seen in neurons under normal conditions.

The formation of mature synapses is a dynamic process with a continuous fluctuation between synapses being formed and unwanted synapses being eliminated [32]. Therefore at any time the number of synapses observed can be considered to be the net result of the number of new synapses formed and those being eliminated. At 7 *DIV *neurons grown on *Fmr1*-/- astrocytes have a significantly greater number of synapses than neurons grown on WT astrocytes. Perhaps at 7 *DIV*, growth with *Fmr1*-/- astrocytes promotes an increase in the production of synapses or a decrease the degree of pruning. A number of studies have suggested that many developmental disorders are caused by a lack of pruning early in development. Our findings therefore support a lack of appropriate synaptic pruning early in development as a potential contributor to the neurological abnormalities seen in FXS.

We found an abnormally high net number of excitatory synapses being maintained in neurons grown on *Fmr1*-/- astrocytes at 7 *DIV*, when compared to neurons grown on WT astrocytes. With time, the difference between the net number maintained in the neurons grown on *Fmr1*-/- astrocytes and those grown on WT astrocytes decreased, until 21 *DIV *at which point there is no longer a significant difference. The neurons grown on *Fmr1*-/- astrocytes have reached a stage of maturity at which their net production of excitatory synapses equals that of neurons grown under normal conditions. Still, there is a possibility that the abnormal early stages of development could play a major role in the ability of neurons to later integrate and transmit information effectively. Abnormal synapses may have been formed alongside atypical local neural networks, and these early alterations may negatively affect the neural circuitry of the region in the long run.

However, from the findings of this study we cannot make conclusions about synapse maturity. It is possible that the increase in synapses observed in the neurons grown on *Fmr1*-/- astrocytes reflects an increased number of immature synapses. Given that the dendritic spine is the site for the majority of excitatory synapses, this finding would be in agreement with numerous studies that identified neurons in Fragile X with an abnormally high number of immature dendritic spines. The methods used in the current study did not permit the assessment of alterations in dendritic spine morphology.

The development of the CNS is a highly organized process requiring the establishment of correct connectivity with specific target cells. The creation of appropriate neural networks requires an intimate coupling of dendritic arborization and synaptogenesis, perfectly timed to promote the normal circuitry underlying normal neurological function. Individuals with FXS syndrome suffer a diverse range of cognitive impairments. Most research has focused on the effects of a lack of FMRP in neurons, and its implications for the underlying neurobiology of FXS. Knowing that astrocytes contribute to the abnormal neurobiology of this disorder, the current results should promote new therapeutic avenues in the treatment of FXS and related autistic disorders.

## Conclusions

Using a co-culture system of neurons seeded on astrocytes, we demonstrated that hippocampal neurons showed delays in dendritic growth patterns and also in the expression of excitatory synapses when interfaced with astrocytes that do not express FMRP. This study reinforces the importance of astrocytes in the regulation of normal neuronal development and in the prevention of an altered dendrite phenotype that characterizes Fragile X and autism. Future experiments will focus on differential expression of astrocyte secreted factors and the role of direct neuronal-astrocyte cell contact in the altered developmental sequences we observed in our tissue culture paradigm.

## Authors' contributions

SJ designed and performed the tissue culture experiments, immunocytochemistry, morphometric analysis and drafted the manuscript. MN assisted with the tissue culture experiments, immunocytochemistry and morphometric analysis. LD conceived the study, participated in its design and helped to draft the manuscript. All authors read and approved the final manuscript.
